# Supplementary material for: Are carbon emissions trading and green financial instruments synergistic? -Comprehensive quantitative research based on content analysis
Source: PLoS One. 2024 Mar 7;19(3):e0298601. doi: 10.1371/journal.pone.0298601 (PMC10919601; doi:10.1371/journal.pone.0298601)
Supplement: S1 Data — (ZIP) [file pone.0298601.s001.zip › Data/地级市Z_F工作报告.zip!/╦─┤¿/─╧│Σ╩╨/└┤╫╘╣1⁄2╓┌║┼í╛┬φ┐╦╩2╛▌═°í┐_2014.docx]

一、2013年工作回顾

　　各位代表：

　　我代表市人民政府，向大会报告工作，请予审查，并请市政协委员和其他列席会议的同志提出意见。

　　一、2013年工作回顾

　　过去一年，面对全球经济复苏疲软和全国全省经济下行压力加大的严峻形势，市政府在省委、省政府和市委的坚强领导下，在市人大、市政协的监督支持下，紧紧围绕建设川东北区域中心城市和成渝经济区北部中心城市的奋斗目标，突出投资拉动、产业支撑、城市发展、民生改善等工作重点，真抓实干，攻坚克难，全市经济社会发展稳中有进、稳中向好。

　　——经济保持健康较快增长。实现地区生产总值1328.6亿元、增长11%，地方公共财政预算收入65.6亿元、增长24%，增速在全省首批经济总量过千亿市（州）中均排列首位；完成社会消费品零售总额523.4亿元、增长14.8%，增速居全省第二。

　　——投资总量突破千亿大关。完成全社会固定资产投资1102.8亿元、增长25.2%，总量在全省地级市中排列首位，增速在全省首批经济总量过千亿市（州）中名列第一。

　　——立体交通网络基本形成。南大梁高速公路南充段建成通车，已建成高速公路8条、通车里程391公里，实现县县通高速。高坪机场年旅客吞吐量突破30万人次。南充港都京作业区多用途码头、旅游客运码头和南充经开区大件码头开港营运，通江达海的黄金水道初步形成。

　　——城市开放度大幅提升。成功举办中国四川国际文化旅游节、中法兰波诗会等国际性文化旅游活动；与法国香槟－阿登大区沙勒维尔－梅济耶尔市签订全面友好合作协议，组团赴法参加第14届国际木偶艺术节，被推选为国际木偶联会常务理事城市。招商引资到位资金533亿元、增长59%，新引进亿元以上项目149个，4家世界500强、5家大型央企、14家上市公司花落南充。

　　——县域经济活力竞相迸发。8个县（市、区）地区生产总值、全社会固定资产投资增速高于全国、好于全省，各县（市、区）地方公共财政预算收入均保持20%以上高位增长。工业园区面积拓展到85平方公里，入园规模以上企业实现产值1304亿元、增长24.7%；鑫达新材料、九州通医药、科创医药等项目开工建设，江苏通光、加加食品、三环电子二期等项目竣工投产。

　　一年来，市政府重点做了以下工作：

　　（一）狠抓枢纽城市建设，发展基础不断夯实。交通枢纽建设加快推进。遂西、巴南广高速公路建设进展顺利；国道212线、318线等国省干线公路维修改造扎实推进，定水经升钟至思依一级公路启动建设，建成通乡通村公路2149公里。兰渝铁路、南充经开区铁路专用线加快建设，汉巴南铁路纳入全省铁路路网规划，遂南广邻城际铁路纳入成渝经济区城际轨道交通线网规划；开通动车25班次，营山成为西部首个动车始发县级城市。高坪机场改扩建民用工程全面完成，新增昆明、西安航线。通讯信息枢纽加快完善。在川东北率先开通4G网络，新增352个行政村通宽带、403个行政村实现光纤接入，成为全国首批信息消费试点城市。区域物流中心加快建设。南充现代物流园基础设施和配套服务设施建设扎实推进，浙江传化等知名物流企业入驻园区并开工建设，阆中、营山等物流节点启动建设，成功申报全省首批现代物流试点示范市和全省区域现代商贸物流中心。

　　（二）狠抓产业城市建设，经济实力稳步增强。工业经济稳中有升。规模以上工业企业增加值增长11.8%；完成技改投资141亿元、增长17.4%。南充经开区列入全省千亿产业园区培育规划，晟达新材料系列项目加快建设，成功招引中机国能、柏林水务等知名企业；九县（市、区）工业园新增入园企业114户，高坪、南部和蓬安工业园列入全省500亿产业园区培育规划。农村经济稳步发展。实现农林牧渔业总产值499.7亿元、增长3.7%，农业增加值297.2亿元、增长3.6%，粮食总产量307万吨、连续11年全省第一。南充国家现代农业示范区成为全省唯一的全国农业改革与建设试点示范区，4个百公里现代循环农业示范带全面建成。9个农业农村综合示范区带动全市新建特色种植业基地50万亩，生猪规模养殖率提高到69%。新建林木基地30万亩，发展林下产业10万亩。升钟水库二期、解元水库、九龙潭水库、油房沟水库等重大水利工程加快推进。“一区八镇九村”统筹城乡改革发展试点工作有序推进。第三产业持续繁荣。实现服务业增加值342.7亿元、增长11%。成功申报丝绸及制品国家外贸转型升级专业型示范基地。“一库两中心”加快建设，成都银行南充分行、乐山市商业银行南充分行以及嘉陵、南部2家村镇银行挂牌开业；仪陇农村信用社组建农村商业银行通过中国银监会审批，营山农村信用社获得省联社同意改制批复。五星花园商圈改造全面完成，顺庆府衙商圈基本形成，一批城市综合体项目加快实施。阆中古城在川东北率先创建为国家5A级旅游景区，全市实现旅游总收入210亿元、增长35.3%。

　　（三）狠抓宜居城市建设，功能品质明显提升。全市城镇化率提高1.6个百分点、达到40.9%；中心城区规模拓展到109平方公里、常住人口达到106万人。规划体系逐步完善。编制完成《南充市生态城市规划》、《南充市城市地下空间开发利用规划》等12个专项规划，全面完成县域新村总体规划。城镇功能不断优化。中心城区北部新城、南部产业新城和下中坝等城市新区加快建设，人民南北中路下穿隧道、林海北路、嘉陵大道建成通车；阆中、仪陇、蓬安跨江大桥加快建设。市档案馆、西充体育馆主体工程完工，南部城市规划馆建成开馆。一批小城镇基础设施进一步完善，23个新农村综合体和930个新村聚居点基本建成。生态环境持续改善。全市新增城市公园21个、城镇绿化景观205万平方米、城市绿道116.3公里；巩固退耕还林成果83.7万亩，森林覆盖率提高到39.3%。西充河流域水质明显改善，生活污水集中处理率和生活垃圾无害化处理率分别达到81.6%、87%，单位生产总值能耗下降4.58%。成功创建全省环境优美示范城市、省级环保模范城市；南部成功创建国家卫生县城，西充成功创建全国绿化模范县。城市管理不断加强。数字城管系统投入使用，中心城区扬尘污染、交通秩序、户外广告和消防隐患等专项治理有效推进，城市秩序进一步规范、形象进一步提升。

　　（四）狠抓幸福城市建设，人民生活持续改善。切实加大民生投入，民生支出占公共财政支出的比重提高到68.3%，“十项民生工程”投入资金105.6亿元、增长20.5%。城镇居民人均可支配收入19206元、增长11.5%，农民人均纯收入7650元、增长13.7%。新增城镇就业7.7万人，城镇登记失业率为4.3%。切实加大扶贫开发力度，13.8万农村扶贫对象脱贫解困。实现新型农村养老保险和城镇居民养老保险制度整合，全市新型农村合作医疗参合率98.9%，城乡居民大病医疗保险试点和城镇补充医疗保险工作有序推进，最低生活保障、临时生活困难救助制度更加完善。开工建设保障性住房3595套、拆迁还房357万平方米，改造棚户区27994户、农村C级危房27870户。新建和改扩建幼儿园153所，建成中小学校舍30万平方米。公立医院改革试点有序推进；川北医学院附属医院新院区、市中心医院下中坝新院区加快建设，市妇幼保健院迁建项目、南充精神卫生中心住院大楼和7个县级急救中心、30个乡镇卫生院全面建成。人口自然增长率为3.12％。。

　　（五）狠抓文明城市建设，社会大局和谐稳定。认真开展“中国梦”主题教育活动，积极推进文明单位、文明村镇、文明集市创建活动，市民素质和城市文明程度不断提升，省级文明城市创建通过省检。成功举办市第二届农民运动会、第四届残疾人运动会、嘉陵江灯戏暨全国地方戏剧节等文化体育活动。民俗音乐《川北婚嫁》、小音乐剧《听树》等艺术作品获国家级大奖。新建7个示范乡镇综合文化站、209个社区书屋，完成1434个行政村广播“村村响”工程和267个农民健身工程。科技对经济增长贡献率达到44%。加强和创新社会管理，高度重视安全生产和信访维稳工作，有序推进食品药品监管体制改革，有力维护公共安全、生产安全，促进社会大局稳定。

　　与此同时，国防动员、人民防空和双拥优抚工作扎实推进，行政监察、审计监督、国资监管、统计调查、检验检疫、科技、工商、质监、海关、气象、档案、保密、国安、邮政、外事侨务对台等工作取得新成绩，民族宗教、地方志、老龄、妇女儿童、残疾人、红十字会等工作发挥了积极作用。

　　各位代表，过去的一年，我们坚持把科学发展作为第一要务，把改善民生作为最大责任，把改革开放作为动力源泉，把县域经济作为重要支撑，在建设区域中心城市的道路上迈出了新的步伐。我们真切感受到，这些成绩的取得，是省委、省政府和市委坚强领导的结果，是市人大、市政协大力支持的结果，饱含着全市干部群众的辛劳与付出，凝聚着社会各界人士的智慧和力量。在此，我代表市人民政府，向全市人民，向各民主党派、工商联、无党派人士和人民团体，向驻市解放军、武警部队官兵、政法干警和各界朋友表示衷心的感谢，并致以崇高的敬意！

　　同时，我们也清醒地认识到，长期积累的结构性矛盾与国内外经济持续下行的压力叠加，影响了去年全市经济预期目标的全面实现。特别是工业投资占比逐年下降，直接影响发展后劲；城乡居民增收形势严峻，尤其是农民收入渠道不多，脱贫解困任务艰巨；个别地方融资成本偏高，债务规模和偿债压力较大。政府职能转变尚未完全到位，行政效能有待进一步提升；个别公务员还存在不务实、不廉洁，甚至违纪违法现象。这些问题，需要我们高度重视，认真研究，切实解决。

　　二、2014年工作安排

　　2014年是全面深化改革的开局之年，也是实施“十二五”规划的关键之年。我国经济正处于“三期”叠加和深度转型的重要关头，经济形势仍然严峻复杂。中央强调必须痛下决心调整经济结构，转变发展方式；省委、省政府要求以经济增长的主动适度换挡为转方式、调结构留出必要空间。我们必须准确把握改革发展大势、国家政策取向和南充阶段特征，统筹考虑稳增长、调结构、促改革、惠民生等因素，充分释放改革动力，挖掘经济增长潜力，保持转型发展定力，全面激发市场活力，促进全市经济持续健康发展。

　　今年政府工作的总体要求是：深入贯彻党的十八届三中全会和中央、省委、市委经济工作会议精神，紧紧围绕率先次级突破、同步全面小康、实现富民强市的总任务，牢牢把握稳中求进、改革创新的核心要求，深入实施“三大发展战略”，着力改革开放、产业支撑、投资拉动、城镇发展和民生改善，全面推进南充经济、社会、城市、生态可持续发展，为建设川东北区域中心城市和成渝经济区北部中心城市奠定更加坚实的基础。

　　全市经济社会发展的主要预期目标是：地区生产总值增长10.5%以上，全社会固定资产投资增长16%以上，地方公共财政预算收入增长16%以上，规模以上工业增加值增长11%以上，社会消费品零售总额增长13.5%以上，城镇居民人均可支配收入增长11%以上，农民人均纯收入增长12%以上，城镇化率提高1.6个百分点以上。

　　实现上述目标，必须重点抓好五方面工作：

　　（一）深化改革开放，有效释放经济发展活力

　　切实抓好重点领域改革。深入推进统筹城乡改革，全面启动农村产权确权颁证工作，探索开展农村承包土地经营权抵押融资和农村土地流转收益保证贷款试点。加快推进金融改革，以优化股权结构和人才结构为核心，着力抓好农村信用社改制工作；支持金融机构开展农村金融服务创新试点；积极推动南充市商业银行上市；探索建立民间资本、地方债券、政策性金融机构等多元可持续的城镇化资金保障机制。深化财税体制改革，强化政府性债务管理和风险防控，推动平台公司发行债券对高利短期债务进行置换；继续做好“营改增”试点工作。不断完善国有资产监督管理体制。积极推行政府购买服务。稳妥推进事业单位分类改革和社会保障、文化教育、科技体制、生态文明制度等方面的改革。深入推进公立医院改革，力争列入第二批公立医院改革国家联系试点城市。

　　不断扩大开放合作。积极开拓海峡西岸、环渤海和东北地区等新的招商区域，主动对接“中外知名企业四川行”等投资促进活动和“西博会”等国际性、区域性展会活动，大力招引龙头企业和配套企业，注重引进企业设立区域总部、研发中心等功能性机构，力争到位资金550亿元以上。争取设立综合保税区、保税物流中心等海关特殊监管区。加强区域交流合作，围绕区域中心城市的功能定位，积极推动区域发展规划、交通基础设施和产业等方面的对接；精心举办“2014四川（南充）·法国周”活动。大力实施“嘉陵江英才工程”，启动首批招才引智计划。做好“4·20”芦山地震灾区对口援建工作，加快推进“一村一街一园”建设。

　　（二）强化产业支撑，大力推进集约集群发展

　　狠抓工业提质增效。加快传统产业改造升级，推进东风南充公司主辅分离，扩大天然气整车和发动机生产能力；启动建设新能源汽车产业园。深化与中丝集团的产业联盟合作，推动丝纺服装产业做大规模、升级换代。支持太极制药搬迁扩能，加快香雪制药、科创医药和九州通医药等企业建设，确保康美药业竣工投产，加速形成医药产业集群。支持轻工食品企业提升产品档次，拓展销售市场。大力培育以新能源、新材料为重点的新兴产业，积极招引电子终端、通讯器材等生产企业，加快建设聚酯、尼龙和鑫达新材料、江苏通光二期等项目，开工建设南充经开区精细化工产业园。大力实施创新驱动发展战略，依托我市科教、人才优势，规划建设创新驱动发展试验区，启动建设大学科技园、大学生创业园，支持潆华工业园申报省级高新区。鼓励各县（市、区）工业园区加快发展，争创省级开发区。

　　推进现代农业发展。大力推进南充国家现代农业示范区建设，连片推进“5+3”特色优势产业基地建设，巩固提升4个百公里现代循环农业示范带。争创以西充为核心区的国家级农业科技园区。加快建设有机农产品生产基地，规划建立区域性有机农产品交易平台。积极创新农田水利设施建设和管护机制，加大高标准农田建设力度，不断提高农业科技应用和机械化水平。全面落实国家粮食安全战略，确保粮食总产量稳中有升。鼓励发展各类农民专业合作社和混合所有制农业产业化龙头企业，密切与农户的利益联结关系。探索建立农村综合产权流转交易平台，鼓励农村土地承包经营权向专业大户、家庭农场、农民合作社、农业企业流转，积极推进适度规模经营。大力发展主体多元、形式多样、竞争充分的社会化服务，不断健全农业社会化服务体系。

　　积极发展现代服务业。加快提升南充现代物流园承载能力，大力招引知名物流企业，争创全国物流示范园区。以“一库两中心”为龙头，着力打造金融一条街，加快建设北部新城金融中心；积极招引更多全国性金融机构进驻南充，力争融资租赁公司、信托投资公司等新型金融业态实现零的突破。开工建设南充国际商贸城，加快建设江东新区、嘉陵新城等商业副中心。大力发展会展经济，力争成为全省次级会展中心。积极推广连锁经营、电子商务等现代营销方式，大力培育健康、养老、文化、信息等新的消费热点。充实提升阆中古城5A级景区内涵，大力支持朱德故里景区创建5A级景区，加快推进金城山旅游景区、嘉陵江第一曲流度假区等景区景点建设。力争服务业增加值增长11%以上。

　　（三）注重投资拉动，不断增强转型发展后劲

　　加大产业投资力度。坚持把扩大工业投资作为优化投资结构的重中之重，力争完成投资280亿元、增长20%以上；支持企业加大技术改造和技术创新投入，力争完成技改投资167亿元、增长18%以上；积极搭建工业投融资平台，有效整合各类资源，筹集工业发展资金。充分发挥财政资金引导作用，鼓励和带动工商资本到农村发展适合企业化经营的现代种养业，保障金融机构农村存款主要用于农业农村，促进“三农”投入明显增长。扩大现代服务业投资规模，加快发展生产性服务业，支持南充现代物流园发行企业债。坚持招大引强，优化商业地产布局，把握供地节奏，加强预售监管，引导房地产业健康有序发展。

　　稳定政府性投资增长。主动对接国家政策支持方向和投资结构调整方向，重点围绕保障性安居工程、重大基础设施、节能环保、信息消费、公共服务等领域，全力争取一批投资额大、带动力强、影响长远的重大项目。加快推进政府融资平台转型升级，进一步拓宽融资渠道，增强融资能力。抓好重大基础设施项目建设，加速推进兰渝铁路、南充经开区铁路专用线建设，扎实做好汉巴南铁路和遂南广邻城际铁路前期工作。加快建设遂西、巴南广高速公路，开工建设南绵高速公路，力争开工建设营达高速公路和南充过境高速公路东北段，有序推进南泸、绵阆巴、营蓬仪阆等高速公路前期工作。启动顺庆经蓬安到营山、长乐至龙蟠等一级公路和西充至嘉陵快速通道建设。开工建设火车北站、嘉陵江航运配套工程、高坪机场航站楼改扩建工程。启动建设金鸡沟水库，加快建设升钟水库二期、油房沟水库，确保解元水库全面建成，九龙潭水库主体工程完工。加大拆迁还房建设力度，开工407万平方米、续建570万平方米、建成163万平方米。

　　充分激发民间投资活力。制定投资领域负面清单，推动民营资本进入金融、通讯、市政设施、保障性住房等领域。鼓励发展混合所有制经济，支持民营资本参股国有企业。推行工商注册制度便利化，放宽注册资本和企业住所限制，逐步改实缴登记制为认缴登记制，变先证后照为先照后证。积极搭建小微企业孵化平台，支持高校毕业生、返乡农民工、科技人才等群体投资创业，加快形成全民创业热潮。

　　（四）坚持以人为本，着力提升城镇发展质量

　　积极推进农业转移人口市民化。全面放开县城和建制镇落户限制，有序放开中心城区落户限制，逐步实施流动人口居住证制度。稳步推进基本公共服务常住人口全覆盖，统筹解决转户居民城镇养老、医疗、教育等问题。继续开展“农民工住房保障行动”，规划建设面向农民工出租的住房小区。健全农民工就业服务体系，完成农民工在岗培训3.6万人。

　　不断优化城镇布局。坚持“全域南充”理念，加快完善“1+3+6+N”的市域城镇体系。稳步扩大中心城区规模；支持南部撤县设市、西充撤县设区，阆中、仪陇、营山、蓬安拓展县城规模；加快发展一批有历史记忆、地域特点、丘区特色的美丽城镇，突出抓好省级、市级重点（试点）镇建设，支持符合条件的乡撤乡设镇、镇改设街道办事处；开展村庄人居环境整治，完成10个新农村综合体、350个新村聚居点建设，促进城镇化与新农村建设协调发展。

　　加快完善城镇功能。大力推进中心城区和各县（市）城市新区建设，不断完善基础设施和公共服务设施。开工建设都京港大桥、将军路大桥等跨江大桥，加快建设金融大道、高都路南延线等城市干道，统筹实施地下管网改造和电缆下地工程。开工建设市规划展览馆、科技馆、博物馆和文化馆，建成南部奥体馆、西充文博馆。启动实施“引嘉入西”北部新城水环境综合治理工程，支持营山实施嘉陵江引水工程，加强中小河流域治理。加快建设中心城区城北公园、东湖公园、南湖公园和仪陇离堆文化公园等城市公园。抓好天然林保护、退耕还林、水土保持等工程建设。加强大气污染治理，改善城区空气质量。全面推进重点领域节能降耗和农村面源污染治理，加快污水、垃圾处理设施建设，确保单位GDP能耗和污染物排放总量稳步下降。充分发挥数字城管综合平台作用，加快建设城区智能交通管理系统，不断提高城市精细化、智能化管理水平。

　　（五）突出民生优先，加快建设和谐幸福家园

　　大力实施民生工程。坚持公共财政的民生投向，力争民生支出占公共财政支出的比重提高到70%。全面实施“十项民生工程”，重点抓好20件民生实事：全部免除义务教育阶段学生作业本费；免除5.06万名家庭经济困难高中学生学费；减免1.64万名家庭经济困难在园幼儿保教费；全面取消县级公立医院药品加成；实行免费婚前体检；提高城乡居民基本医疗保险水平；提高城乡居民社会养老保险水平；新建改造7800张养老床位，建设120个城乡社区日间照料中心；资助68623名重度残疾人护理费用；对初次创业并吸纳3名以上劳动者就业的高校毕业生一次性给予1万元补贴；建设10.7万套城镇保障性住房；将30%公共租赁住房定向出租给农民工；改造2万户农村危房；解决37万农村人口和73019名学校师生安全饮水问题；改善150公里乡村公路，建设600公里公路安全护栏；实施3121个村公共服务设施运行维护补助；继续加大西充河流域综合治理力度；新（改）建专业蔬菜基地4.5万亩，建成鲜活农产品便民店（超市）50个；完成20所乡镇卫生院和100个村卫生室建设；新建庄稼医院50个、农村社区综合服务社50个。

　　完善社会保障体系。实施就业优先战略，确保新增城镇就业8万人以上。完善基本养老、医疗保险关系转移接续办法，积极推进城乡居民大病医疗保险试点，全面实施困难群众重特大疾病医疗救助。健全最低生活保障、临时生活困难救助等社会救助体系，提高群众受益面和保障水平。大力实施扶贫开发5年规划，着力提高扶贫精准度，确保34.4万人脱贫解困。

　　不断强化公共服务。继续加大教育投入，改造义务教育阶段薄弱学校76所，完成中小学校舍建设14.6万平方米；加快实施中心城区基础教育布点规划，开工建设北部新城高中；大力发展职业教育，支持发展高等教育，继续加强特殊教育。加快推进市中心医院下中坝新院区一期工程、川北医学院附属医院新院区医技楼和市儿童医院等重大卫生项目建设，进一步完善医疗卫生服务体系。扎实做好国家公共文化服务体系示范区创建工作，节俭务实举办中国（南充）首届国际木偶艺术周、第四届中国（南充）嘉陵江合唱艺术节等文体活动。深化统计改革，抓好市第三次全国经济普查。扎实做好第九届村（居）委会换届选举工作，积极创建第十届省级双拥模范城。

　　切实加强社会治理。创新社会治理体制机制，维护社会公平正义。畅通和规范群众诉求表达渠道，健全行政复议案件审理机制，开展网上受理信访，实行诉讼与信访分离，促进群众合理诉求及时就地解决。深入推进“平安南充”创建活动，创新立体化社会治安防控体系，严厉打击各类违法犯罪行为；提升流动人口和特殊人群管理服务水平，加大依法管理网络力度。深入推进食品药品监管体制改革，加快建立产品可追溯体系和全过程监管制度，保障食品药品安全。夯实安全生产基层基础，加强防灾减灾救灾体系建设和风险管理，坚决遏制重特大安全生产事故发生。

　　三、政府自身建设

　　各位代表，一年来，我们切实加强“五型”政府建设，努力打造人民满意政府。办理市人大代表议案2件、建议267件，政协提案392件，办结率100%。坚持重大决策广泛征求社会各界意见，邀请市民代表351人次列席市政府全体会、常务会和规委会，在全省率先开通市、县两级政务微博发布厅和市政府官方微信。大力推进简政放权，取消和下放行政审批项目61项、非行政许可项目33项，取消行政事业性收费34项，成为全省行政审批事项最少的市（州）之一；大力推行并联审批，实现行政审批再提速40%。坚决抵制“四风”，精简会议35.8%、文件39.6%，“三公”经费支出下降35.2%。

　　新的一年，我们将认真落实中央关于改进作风、密切联系群众的“八项规定”，扎实开展以“为民、务实、清廉”为主要内容的党的群众路线教育实践活动，进一步提升政府的决策力、执行力和公信力。

　　（一）坚持民主施政，搭建公开透明的互动平台。完善重大行政决策咨询论证和公众征询制度，扩大市政府全体会、常务会、专题会和规委会向社会开放程度。建设市民呼叫中心、视频直播系统，开办“政务面对面”电视和网络访谈节目，健全新闻发布制度。大力推进政务公开标准化，加快建设行政权力公开运行平台，实行行政权力网上公开透明运行。

　　（二）坚持务实勤政，强化勇争一流的责任担当。完善政府绩效管理体系，对重点专项工作、政府投资项目、财政资金使用等开展绩效评价。大力倡导创先争优，积极推行部门责任公开承诺制，健全重大项目、重点工作跟踪落实和责任追究机制，形成言必责实、行必责实、功必责实的良好风气。从严控制政府发文和会议数量。大力开展“走基层、解难题、办实事、惠民生”活动，积极为基层群众和企业排忧解难。

　　（三）坚持高效理政，打造服务优良的政务环境。紧紧围绕打造川东北投资首选地的目标，按照“审批项目最少、审批流程最优、审批时间最短”的要求，进一步精简、下放行政审批事项和非行政许可审批事项；深入推进并联审批，建立重大项目审批事项全程代理代办制；完善政务服务体系，加强政务服务标准化建设；扎实开展投资软环境常态治理，着力解决庸懒散软、吃拿卡要等问题，进一步提高机关行政效能和办事效率。

　　（四）坚持依法行政，促进行政权力的规范运行。自觉接受市人大及其常委会的法律监督、工作监督和市政协的民主监督，提高人大代表议案、建议和政协提案办理实效，主动接受群众和舆论监督。坚持用法治思维和法治方式履行职能职责，依法处理各种社会矛盾和问题。加强和改善行政执法，规范行政处罚自由裁量权，充实重点领域基层执法力量。

　　（五）坚持廉洁从政，树立风清气正的良好形象。认真落实党风廉政建设责任制，加强行政监察和审计监督，突出抓好工程建设、土地出让、征地拆迁、政府采购等重要领域和关键环节的监督。加强公共资源交易市场规范化建设，不断完善电子招投标交易平台、公共服务平台和行政监督平台。从严控制“三公”经费支出，严禁新建楼堂馆所，坚决抵制享乐主义和奢靡之风。

　　各位代表，时代激励我们改革创新，责任鞭策我们砥砺前行。让我们高举中国特色社会主义伟大旗帜，在市委的坚强领导下，紧紧依靠和团结全市人民，以踏石留印、抓铁有痕的精神，以迎难而上、奋勇争先的锐气，埋头苦干，开拓进取，为加快建设川东北区域中心城市和成渝经济区北部中心城市，全面建成小康社会而努力奋斗！
